# Availability and readiness of health care facilities and their effects on under-five mortality in Bangladesh: Analysis of linked data

**DOI:** 10.7189/jogh.12.04081

**Published:** 2022-09-17

**Authors:** Nuruzzaman Khan, Nahida Islam Trisha, Mamunur Rashid

**Affiliations:** Department of Population Science, Jatiya Kabi Kazi Nazrul Islam University, Mymensingh, Bangladesh

## Abstract

**Background:**

Under-five mortality is unacceptably high in Bangladesh instead of governmental level efforts to reduce its prevalence over the years. Increased availability and accessibility to the health care facility and its services can play a significant role to reduce its occurrence. We explored the associations of several forms of child mortality with health facility level factors.

**Methods:**

The 2017-18 Bangladesh Demographic and Health Survey (BDHS) data and 2017 Bangladesh Health Facility Survey (BHFS) data were linked and analysed. The outcome variables were neonatal mortality, infant mortality, and under-five mortality. Health facility level factors were considered as major explanatory variables. They were the basic management and administrative system of the nearest health care facility where child health care services are available, degree of availability of the child health care services at the nearest health care facility, degree of readiness of the nearest health care facility (where child health care services are available) to provide child health care services and average distance of the nearest health care facility from mothers’ homes where child health care services are available. The associations between the outcome variables and explanatory variables were determined using the multilevel mixed-effect logistic regression model.

**Results:**

Reported under-five, infant and neonatal mortality were 40, 27, and 22 per 10 000 live births, respectively. The likelihood of neonatal mortality was found to be declined by 15% for every unit increase in the score of the basic management and administrative system of the mothers’ homes nearest health care facility where child health care services are available. Similarly, degree of availability and readiness of the mothers’ homes nearest health care facilities to provide child health care services were found to be linked with 18%-24% reduction in neonatal and infant mortality. On contrary, for every kilometre increased distance between mothers’ homes and its nearest health care facility was found to be associated with a 15%-20% increase in the likelihoods of neonatal, infant and under-five mortality.

**Conclusions:**

The availability of health facilities providing child health care services close to mothers’ residence and its readiness to provide child health care services play a significant role in reducing under-five mortality in Bangladesh. Policies and programs should be taken to increase the availability and accessibility of health facilities that provide child health care services.

The Sustainable Development Goals (SDG) started in 2015 with ambitious targets to reduce the neonatal and under-five mortality rates to 12 and 25 per 1000 live births, respectively, by 2030 [1]. The Millennium Development Goals (MDGs), which ended in 2015 with more than 50% (from 90 to 43 per 1000 live births) reduction of global under-five mortality, helped the world to make such highly ambitious targets [2,3]. However, such an average estimate of under-five mortality as well as reduction during the MDGs does not truly represent the situation of under-five mortality in low- and middle-income countries (LMICs). The rate of under-five mortality in LMICs is still 69 per 1000 live birth- a rate that was the global average on the 2000s [4,5]. Consequently, LMICs represent more than 90% of the 5 million under-five deaths that occur globally, and the probability of under-five mortality is 14 times higher in LMICs than in developed countries [5].

The rate of under-five mortality is even higher in Asian and African regions where around 80% of the global under-five deaths occur, though these two regions account for only 53% of the global births [6]. The causes of such higher under-five mortality in LMICs, in particular Asian and African regions, are infectious diseases including pneumonia, diarrhoea and malaria as well as pre-term birth complications, birth asphyxia and trauma, and congenital anomalies [5,7]. Cost-effective, proven interventions, including quality of maternal health care services around the time of birth and child health care services, exist to prevent and treat infectious diseases and other causes associated with under-five mortality [5]. This requires trained and equipped health workers, including midwives and the availability of essential commodities [6].

Bangladesh contributes around 4.30% of the total live births in the South Asian region with around 3 million yearly live births [8]. However, the country is ranked number 4 in terms of neonatal mortality in South Asia, whereas Pakistan, Afghanistan and India are ranked in the first three, respectively [5]. Such a higher rate of child mortality in Bangladesh is reported instead of its significant progress in preventing infectious diseases, including diarrhoea and pneumonia, increasing breastfeeding practice and immunization, improvement of maternal undernutrition, reduction in adolescent pregnancy, and a significant increase in maternal health care services use [8-11]. A similar progress has been reported in other South Asian countries as well as LMICs [12-15]. Consequently, most of LMICs, including Bangladesh are currently off-track in achieving the SDGs targets related to child mortality [16,17].

Healthcare facilities in Bangladesh and other LMICs are often criticized for the poor quality of available services, and lack of health care personnel and equipment [11]. However, a significant progress has been achieved over the decades, particularly during the MDGs [2]. The pace of progress is even now much faster than before to ensure the universal health coverage in line with the SDGs’ target [18]. Therefore, it is desirable that such progress is now making a significant contribution in reducing child mortality, though research to justify this understanding is lacking in Bangladesh and other LMICs. The reason for such lacking is the absence of longitudinal data as well as linked individual and health care facility level data. Consequently, available research mainly explored the socio-demographic and behavioural factors associated with child mortality, including maternal age and education, under-nutrition, and poor feeding practice where effects of health care facility level factors were ignored [19-23]. As the occurrence of child mortality is interconnected with multidimensional factors, including factors at individual-, household-, community-, and healthcare-facility level, therefore, determining such associations without considering the health care facility level factors may produce imprecise findings.

To address these limitations, we conducted this study to explore the association between health care facility level factors and child mortality in Bangladesh adjusting for individual-, household-, and community level factors.

## METHODS

The 2017/18 Bangladesh Demographic and Health Survey (BDHS) and 2017 Bangladesh Healthcare Facility Survey (BHFS) were linked and analysed in this study. Details of sampling procedures of these surveys are available in the respective survey reports [24,25].

The 2017/18 BDHS is the nationally representative household survey conducted as part of the Demographic and Health Survey Program of the USA. The survey used a validated questionnaire to collect data and received ethical approvals from the ICF International, USA and the Ministry of Health and Family Welfare of Bangladesh. The National Institute of Population Research and Training (NIPORT) conducted this survey along with the Mitra and Associates (a private research firm). The survey selected nationally representative households through two-stage stratified random sampling methods. At the first stage of the sampling, the survey selected a list of 675 clusters, covering each division and urban/rural area of Bangladesh. The clusters were selected from a list of 293 579 clusters of Bangladesh which was generated by the Bangladesh Bureau of Statistics as part of the 2011 Bangladesh National Population Census. A total of 672 clusters was retained finally after excluding 3 clusters because of the extreme flood. A fixed number of 30 households was selected at the second stage from each selected cluster. Finally, the survey was conducted in 19 457 households with an over 96% inclusion rate. There were 20 376 women in these selected households, of them, 20 127 women were interviewed with a response rate of 98.8%. Informed written consent was obtained from all participants [25].

The 2017 BHFS was also conducted as part of the Demography and Health Survey program of the USA. The survey collected data from 1524 health care facilities selected proportionately from the public, private and non-government sectors. Both census and stratified random sampling methods were used to select these facilities from the 19 811 registered health care facilities in Bangladesh.

The GPS point locations are available for each of the 672 PSUs included in the 2017 BDHS and 1524 health care facilities included in the 2017 BHFS. We linked the PSUs with the nearest health care facilities using the administrative boundary linkage method. The details of this method have been published elsewhere [26].

### Analytical sample

Conditions applied to be included in this study were: (i) the women who were given at least one live birth within five years preceding the survey and (ii) recorded survival status of the corresponding children. A total of 8759 women met these criteria, as such they were analysed in this study.

#### Outcome variable

We considered three outcome variables: neonatal mortality (death occurred within 1 month of live birth), infant mortality (deaths occurred within 12 months of live birth) and under-five mortality (death occurred within 60 months of live births). The BDHS recorded these mortality data by asking women whether they had any live birth within five years of the survey date and survival status of their respective children. In the occurrence of more than one live birth within five years, survival status data were collected for every child. These data were then recategorized by following the relevant guidelines of the WHO to generate the outcome variables [27].

#### Explanatory variables

Health facility level factors were our primary explanatory variables. Several health facility level factors were considered, including basic management and administrative system of the nearest health care facility where child health care services are available, degree of availability of child health care services at the nearest health care facilities, readiness of the nearest health care facility (where child health care services are available) to provide child health care services and average distance on road communication from mother’s resided cluster to the nearest health care facility where child health care services are available. The variables used to generate scores for these corresponding variables are presented in Table S1 in the **Online Supplementary Document**. A total of seven variables was used to generate score for the variable “basic management and administrative system of the nearest healthcare facility where child healthcare services are available”. We considered 35 variables to generate the score for the degree of availability of child health care services at the nearest health care facilities. Total of 10 variables were considered to generate the corresponding score for the readiness of the nearest health care facility (where child health care services are available) to provide child health care services. For generating scores, we have given “1” point if the service referred by that particular variable was available in the health care facility and “0” was given for non-availability. The combined score for each item considered under the particular dimension was then generated by adding the corresponding individual score. The average distance on-road communication of the nearest facility providing child health care services was also calculated and included as a health facility-level variable. The cluster nearest child health care facility was identified first. Then using Bangladesh road communication data, the average distance from the cluster to its nearest health facility was calculated [28].

Other explanatory factors were selected in two stages. At first, we generated a list of all possible confounders by reviewing the relevant studies conducted in LMICs [4,5,20,21,29-32]. Availability of the selected variables was then checked in the data set we analysed. The variables available in the data set were then considered together with considered health care facility level factors and forward regression models were run. The variables found insignificant at 0.20 or more were deleted. Multicollinearity was also checked. Factors that were finally selected were then categorized as individual-, household-, and community level factors and included in this study. Individual level factors were maternal age at birth, maternal education, and maternal formal employment status. Households level factors were partner’s educational status, sex of the household’s head, total children ever born, exposure to mass media, and wealth quintile. Place of residence and region of residence were considered as community level factors.

### Statistical analysis

Descriptive statistics were used to summarise the characteristics of the respondents. Multilevel mixed-effect logistic regression model was used to assess the associations between child mortality and health facility level factors adjusted for individual-, household-, and community-level factors. The reason for using the multilevel mixed-effect logistic regression model was hierarchical structure of the BDHS data, in which individuals are nested within a household and households are nested within a cluster. This creates multiple dependencies in the data for which multilevel mixed-effect logistic regression is deemed the appropriate approach [33]. Both unadjusted and adjusted models were run. Results are reported as odds Ratios (OR) with 95% confidence intervals (95% CI). Statistical analyses were conducted using Statistical package R (version 4.10).

## RESULTS

Background characteristics of the respondents are presented in **Table 1**. Mean age of the respondents was 24 years and mean years of education was 6.7 years. Mean age of the children was 29.1 months. Around 48% of the total under-five children were girls.

**Table 1:** Key information about the study participants and outcome variables

Demographics of mothers	Frequency (n = 8759)	Estimate
Mean age in years (mean±SD)	8759	24.0 (±5.5)
Mean weight in kilograms (mean±SD)	8623	51.7 (±10.2)
Mean years of education (mean±SD)	8759	6.7 (±3.7)
**Demographics of under-five children**		
Mean age in months (mean±SD)	8759	29.1 (±17.6)
Girls, prevalence (95% CI)	8759	47.7 (46.6-48.9)
**Outcomes**		
Neonatal mortality per 1000 live births, prevalence (95% CI)	189	21.6 (18.5-25.3)
Infant mortality per 1000 live births, prevalence (95% CI)	239	27.2 (23.6-31.3)
Under-five mortality per 1000 live births, prevalence (95% CI)	351	40.1 (35.6-45.2)

SD –bstandard deviation, CI – confidence interval

We explored the distribution of neonatal, infant and under-five mortality across the considered explanatory variables. Relevant results are presented in **Table 2**. Occurrence of neonatal mortality was found higher among mothers aged ≥35 years, primary educated mothers, mothers not working, mothers whose partners were uneducated, mothers who resided in a household whose head was female, mothers who had two or more children at the time of the survey, place of residence was urban, and place of region was Barishal and Rangpur. A similar pattern was observed for infant and under-five mortality, except for the place of region while infant and under-five mortality were found higher in the Sylhet division.

**Table 2:** Distribution of neonatal, infant and under-five mortality across background characteristics, Bangladesh, 2017/18

Characteristics	Neonatal mortality per 1000 (95% CI)	Infant mortality per 1000 (95% CI)	Under-five mortality per 1000 (95% CI)
**Maternal age at birth**			
≤19	22.8 (16.1-32.1)	26.0 (18.9-35.5)	47.4 (37.8-59.2)
20-34	21.1 (17.3-25.5)	27.6 (23.3-32.7)	38.4 (33.1-44.4)
≥35	25.1 (12.6-49.5)	28.2 (14.7-53.3)	29.2 (15.6-54.1)
**Mother’s educational status**			
No education	25.6 (15.7-41.6)	32.1 (20.3-50.4)	52.6 (35.6-77.1)
Primary	27.2 (20.5-35.9)	36.0 (28.2-45.7)	48.1 (39.2-58.9)
Secondary	19.3 (14.9-25.0)	24.0 (19.1-30.0)	38.4 (32.3-45.6)
Higher	16.7 (10.0-27.6)	18.8 (11.7-30.0)	24.5 (16.3-36.7)
**Mother’s working status**			
Yes	24.1 (19.1-30.3)	28.9 (23.5-35.5)	40.1 (35.6-45.2)
No	19.8 (15.87-24.8)	26.0 (21.4-31.5)	39.5 (33.9-46.0)
**Partner’s educational status**			
No education	24.8 (16.1-38.0)	33.8 (23.8-47.7)	48.9 (36.5-65.3)
Primary	21.2 (16.0-28.0)	27.2 (21.2-34.9)	40.1 (32.6-49.1)
Secondary	21.7 (16.1-29.2)	28.1 (21.6-36.6)	42.6 (34.5-52.5)
Higher	17.9 (11.6-27.6)	18.8 (12.4-28.5)	27.4 (19.7-38.0)
**Sex of the household’s head**			
Male	20.9 (17.6-24.9)	27.0 (23.2-31.5)	40.8 (35.9-46.2)
Female	26.2 (17.4-39.3)	28.7 (19.2-42.5)	36.1 (25.5-50.9)
**Total children ever born**			
≤2	14.3 (11.2-18.2)	16.8 (13.6-20.9)	32.7 (27.8-38.5)
>2	36.6 (29.2-45.8)	48.5 (40.0-58.6)	55.3 (46.0-66.3)
**Exposure to mass media**			
Little exposed	20.9 (16.3-26.9)	28.0 (22.4-35.1)	43.7 (36.0-53.0)
Moderate exposed	14.9 (10.3-29.9)	20.8 (13.7-31.5)	39.0 (32.8-46.3)
Highly exposed	24.0 (19.3-29.9)	28.5 (23.3-34.8)	35.5 (25.8-48.6)
**Wealth quintile**			
Poorest	23.2 (17.1-31.3)	29.5 (22.7-38.3)	48.4 (38.6-60.5)
Poorer	21.4 (14.9-30.7)	29.8 (22.2-39.9)	39.9 (31.0-51.1)
Middle	23.4 (16.7-32.6)	26.4 (19.3-36.0)	36.4 (28.1-46.9)
Richer	21.7 (14.5-32.5)	27.9 (19.4-40.1)	41.4 (30.7-55.6)
Richest	18.4 (11.9-28.2)	22.1 (14.8-32.7)	33.5 (24.4-45.9)
**Place of residence**			
Urban	25.8 (19.2-34.5)	30.6 (23.3-40.0)	43.7 (35.0-54.4)
Rural	20.1 (16.7-24.1)	26.0 (22.0-30.6)	38.8 (33.6-44.7)
**Place of region**			
Barishal	26.1 (16.7-40.6)	32.0 (22.2-45.9)	46.1 (34.5-61.2)
Chattogram	23.4 (16.8-32.5)	25.5 (18.2-35.5)	36.4 (27.5-48.0)
Dhaka	21.3 (14.7-30.8)	27.2 (19.5-38.0)	41.4 (31.3-54.7)
Khulna	19.7 (12.5-30.7)	25.5 (17.4-37.2)	34.0 (24.1-47.7)
Mymensingh	12.8 (7.3-22.4)	17.2 (10.8-27.3)	34.1 (25.8-44.8)
Rajshahi	17.2 (0.9-30.1)	24.9 (15.3-40.4)	42.3 (28.7-62.2)
Rangpur	29.4 (19.5-44.2)	30.9 (20.8-45.7)	39.4 (26.7-57.8)
Sylhet	22.7 (14.7-34.8)	39.2 (29.5-45.7)	52.3 (40.7-67.1)

**Table 3** presents the association of child mortality with health facility level factors that were determined using the multilevel mixed-effect logistic regression model. Both unadjusted and adjusted models were run separately for each of the three outcome variables: neonatal mortality, infant mortality, and under-five mortality. We found significant effects of health care facility level factors on each of the neonatal mortality, infant mortality, and under-five mortality in the adjusted model. The associations were mostly persists following the adjustment of the individual-, household-, and community level factors in the adjusted model. The likelihoods of neonatal mortality was found to be declined by 15% (aOR = 0.85, 95% CI = 0.63-0.99) and 16% (aOR = 0.84, 95% CI = 0.62-0.96) for every unit increase of the scores of the basic management and administrative system of the nearest health care facility where child health care services are available and the degree of availability of child health care services at the nearest health care facility, respectively. Similarly, per unit increase in the score of readiness of the nearest health care facility (where child health care services are available) to provide child health care services was found to be associated with a 32% (aOR = 0.68, 95% CI = 0.47-0.90) reduction of neonatal mortality. In contrary, for every kilometre increase in distance of the nearest health care facility from mothers’ homes where child health care services are available was found responsible for 1.20 times (aOR = 1.20, 95% CI = 1.01-1.45) increase of neonatal mortality.

**Table 3:** Multilevel mixed-effect logistic regression models assessing the relationships of several forms of child mortality with health facility-level factors adjusted for individual-, household-, and community-level factors (n = 8759)

Characteristics	Neonatal mortality, OR (95% CI)	Infant mortality, OR (95% CI)	Under-five mortality, OR (95% CI)
**Unadjusted association**			
Basic management and administrative system of the nearest health care facility where child health care services are available	0.80 (0.60-0.98)*	0.89 (0.68-1.08)	0.96 (0.80-1.84)
Degree of availability of child health care services at the nearest health care facility	0.63 (0.43-0.85)*	0.82 (0.68-0.99)*	0.90 (0.54-1.29)
Readiness of the mothers’ homes nearest health care facility (where child health care services are available) to provide child health care services	0.64 (0.45-0.86)*	0.68 (0.38-0.98)*	0.72 (0.40-0.95)*
Average distance on road communication from mothers’ resided cluster to the nearest health facility	1.28 (1.03-1.55)*	1.18 (1.01-1.59)*	1.13 (1.01-1.33)*
**Adjusted association†**			
Basic management and administrative system of the nearest health care facility where child health care services are available	0.85 (0.63-0.99)*	0.89 (0.68-1.08)	0.95 (0.78-1.86)
Degree of availability of child health care services at the nearest health care facility	0.66 (0.45-0.89)*	0.82 (0.68-0.99)*	0.93 (0.56-1.30)
Readiness of the mothers’ homes nearest health care facility (where child health care services are available) to provide child health care services	0.68 (0.47-0.90)*	0.68 (0.38-0.98)*	0.75 (0.41-0.96)*
Average distance on road communication from mothers’ resided cluster to the nearest health facility	1.20 (1.01-1.45)*	1.18 (1.01-1.59)*	1.15 (1.04-1.36)*

OR – odds ratio, CI – confidence interval

*P < 0.01.

†Models are adjusted for maternal age at birth, maternal education, maternal formal employment status, partner’s educational status, sex of the household’s head, total children ever born, exposure to mass media, wealth quintile, place of residence and region of residence.

Similarly, one unit increase in the scores of the degree of availability of child health care services at the nearest health care facility and readiness of the nearest health care facility (where child health care services are available) to provide child health care services were found to be associated with a 18% (aOR = 0.82, 95% CI = 0.68-0.99) and 32% (aOR = 0.68, 95% CI = 0.38-0.98) reductions of infant mortality, respectively. Moreover, one kilometre increase of the nearest health care facility where child health care services are available from mothers’ homes was found to be associated with 1.18 times (aOR = 1.18, 95% CI = 1.01-1.59) increase in infant mortality. One unit increase score of the of readiness of the nearest health care facility where child health care services are available to provide child health care services was found associated with a 25% (aOR = 0.75, 95% CI = 0.41-0.96) reduction in under-five mortality. In comparison, under-five mortality was found to be increased by 15% (aOR = 1.15, 95% CI = 1.04-1.36) for every unit increase in the average distance of the nearest health care facility from mothers’ homes where child health care services are available.

## DISCUSSION

The aim of this study was to explore the associations of several forms of child mortality with health care facility level factors. Four health care facility level factors were considered which were generated covering management and administrative system of the mothers’ home nearest health care facility where child health care services are available, degree of availability of child health care services at the nearest health care facility degree of readiness of the mothers’ homes nearest health care facility (where child health care services are available) to provide child health care services, and average distance on road communication from mothers’ resided cluster to the nearest health facility. On average, increasing scores of the health facility level factors were found protective to the occurrence of neonatal, infant, and under-five mortality. In addition, around 15%-20% increase in the occurrence of neonatal, infant, and child mortality were reported for every kilometre increase in the distance of the nearest health care facility providing child health care services from mother’s homes. These findings are robust as they were generated by analysing the two nationally representative data sets and adjusted for possible confounders. The findings will help the policymakers to know the area where focus is needed to face ongoing challenges of under-five mortality and in designing policies and programs, accordingly.

There is a common understanding that inadequate health care services and poor quality of available services have a negative effect on child health, including child morbidity and mortality [34,35]. However, when this comes to specific dimensions, such as particular area of inadequacy, available research in LMICs are mostly limited and they cannot answer these things. As far as we know, for the first time in LMICs, we considered health care facility level factors, including management and administrative system of the mothers’ homes nearest health care facility, degree of availability of the child health care services at the mothers’ homes nearest health care facility and its readiness to provide child health care services and determined their associations with neonatal, infant and under-five mortality. As such we could not validate our findings. However, consideration of such specific wings will help the policymakers to know the areas that need to be prioritized.

In this study, poor administrative and management system, lower availability of the child health care services and lower readiness of the health care facility to provide child health care services were found to be linked with higher likelihoods of neonatal, infant and under-five mortality. There may be direct and indirect pathways of such associations. Availability of health care services and quality of health care services in the health care centre depend on the proper linkage among several wings of the health care facility [36,37]. This includes administrative and management systems, availability of the health care personnel and health care equipment and their readiness to provide health care services [24]. Poor performance in any wing, therefore, affects performance in other wings too, as such indirectly affects the overall performance of the health care facility [24]. For instance, the functional availability of the health care providing equipment does not always mean its readiness to provide services [38-40]. This may arise because of lack of skilled health care personnel to operate equipment and/or lack of proper initiative from the administrative system to ensure use of the equipment and vice versa [40]. This is particularly an issue in Bangladesh as well as other LMICs where health care facilities are often run with either inadequate equipment or the health care personnel, or both [41,42]. Moreover, development activities in the health care facility are often not representative of the community level requirement [43]. This concern is particularly true for the rural area, from where around 75% of the study population were included, where there are no/limited alternative in choosing the health care facilities [41]. In addition, health care facilities in urban area often do not focus on specific requirements for the disadvantaged urban population (eg, slums population) though they represent almost half of the total urban population and mostly depend on the government health care facility for seeking services. This dependency comes from higher expense in the private health care facility in urban area which mostly disadvantaged population could not bear. Consequently, this group observes a higher rate of child mortality, which further influence overall urban estimate, as reported in this study as well as a recent study in Bangladesh [31].

In Bangladesh, as like other LMICs, governmental health care facility plays a driver role in providing child health care services, particularly for complicated cases [38,41]. The reasons are: (i) availability of the health care facility at lower distance, (ii) free or less expensive health care services and (iii) availability of the health care personnel and equipment [38,42]. However, health care facilities in Bangladesh are not equally equipped and the services available there are not consistent [41]. For instance, health care services for complicated cases are only available at the district or divisional level hospitals [11]. Basic services are available at the upazila (sub-unit of district) and community level hospitals. Consequently, people need to travel to district or divisional level hospitals to access health care services in complicated cases for their children. This could increase the occurrence of child mortality as many people do not have the financial capacity to travel to district/divisional level hospitals, buy medicines and stay there for accessing services [44,45]. Even if people have this capacity, transportation is still an important issue considering, (i) transportation to go to the district or divisional level hospitals may not be available particularly for the rural area, and (ii) district or divisional level facilities may stay far and it may take longer hours to go to the health care facility [44,45]. This understanding is consistent with this study’s findings of a 15%-20% increase in under-five mortality for every kilometre increased health care facility nearest to the maternal homes where child health care services are available. A similar association has also been reported in other studies in LMICs [44,46,47].

We found the association of health care facility level factors with neonatal mortality was strongest than association of health care facility level factors with infant mortality and under-five mortality. Moreover, increase scores of the management and administrative system of the mothers’ homes nearest health care facilities and degree of services availability of that health care facility were found associated with reduced odds of neonatal and infant mortality, but not for the under-five mortality. These associations further support the distance and quality issues of the services are played important roles in the occurrence of under-five mortality. The underlying mechanism could be explained by the children’s immune power which is increased with the increased age of children [48]. As such, children who are comparatively high at age have usually better immune power. Consequently, hospital admission is comparatively lower among higher-aged children as compared to the new-born because they mostly get recovered from diseases with minimal treatments available at the community level [49]. Even for complicated cases, such better immune power of the comparatively higher aged children keep them survive for a moderately higher time, as such their parents get a chance to bring them to the health care facilities, even it is located in far [45]. An opposite direction is reported for neonatal and infant mortality as they have comparatively poor immune power, therefore parents may not get enough time to bring them to the health care facility.

The implications of our findings are that there needs to be substantial investment in the health care sector to ensure availability of the child health care services at the community level health care facilities, such as community clinic. However, health care facilities where child health care services are available are usually located in urban areas and rural health care facilities are often under-resourced and are not capable of providing the child health care services. The reasons are lack of skilled health care personnel at the community level health care facilities, poor or under-developed infrastructure and lack of medical equipment. Unchanged government budget allocations in health care sectors over the years (2.02% (2000) and 2.37% (2016) of gross domestic product) in the face of rising health care demands are often causes of such crisis [50]. Transportation services in the urban health care facilities to bring children who need critical care to the urban health care facilities are also lacking. Therefore, priority also needs to be given to improving the transportation facilities of the health care facilities. This needs universal health care coverage- a target that is also present in the SDGs to be achieved by 2030 [1]. Consequently, the focus is needed on the governmental level policies and programs.

This study has several strengths and some limitations. The most important strength of this study is the analysis of two nationally representative household and health care facility surveys data. By this, the associations of health care facility level factors with several form of child mortality were determined. As far as we know this sort of understanding is first in Bangladesh and other LMICs. Moreover, findings were adjusted with the range of adjustment factors at the individual-, households-, and community- level factors. Advanced statistical modelling was used to determine the associations. Therefore, the findings of this study are robust and could be applicable for the national level policy and program making. However, since we analysed a cross-sectional household survey data, the findings are correlational only, not casual. To secure the privacy of the respondents, BDHS displaced clusters’ locations up to 5 kilometres for rural areas and 2 kilometres for urban areas. Therefore, the calculated average distance of the nearest health facility could be slightly different from the actual distance. However, the BDHS ensured that the new perturbed locations fell within the designated administrative boundaries. Therefore, errors from displacement are likely to be random and minimum. A previous study found that the effect of this variation is insignificant [51]. Regardless of these limitations, to our knowledge, this is the first study in the context of LMICs and Bangladesh that examined the influence of health facility-level factors on under-five mortality adjusted for individual-, household-, and community-level factors. Therefore, the finding of this study is likely to be precise and can be generalisable in countries with similar features to Bangladesh and may help in making evidence-based policies and programs.

## CONCLUSIONS

We reported prevalence of under-five, infant and neonatal mortality rates in Bangladesh were 40, 27 and 21 per 1000 live births. Increase score on the basic management and administrative system of the nearest health care facility where child health care services are available was found negatively associated with the occurrence of neonatal mortality. In addition, degree of availability of child health care services at the nearest health care facility and readiness of the nearest health care facility (where child health care services are available) to provide child health care services were found negatively associated with neonatal and infant mortality. Average distance on road communication from mothers’ resided clusters to the nearest health facility was found to be linked with the 15%-20% increase in the likelihoods of neonatal, infant and under-five mortality. Healthcare facilities should be strengthened to provide child health care services, particularly in the rural area where transportation is an ongoing issue. Healthcare facilities’ transportation facilities, including ambulance services, should also be strengthened.

## Additional material


Online Supplementary Document

## References

[1] Robert KW Parris TM Leiserowitz AA What is sustainable development? Goals, indicators, values, and practice. Environment. 2005;47:8-21.

[2] Beattie RM Brown N Cass H Millennium development goals progress report. Archives of Disease in Childhood. 2015;100:S1. 10.1136/archdischild-2014-307933 25613958

[3] Fullman N Barber RM Abajobir AA Abate KH Abbafati C Abbas KM Measuring progress and projecting attainment on the basis of past trends of the health-related Sustainable Development Goals in 188 countries: an analysis from the Global Burden of Disease Study 2016. Lancet. 2017;390:1423-59. 10.1016/S0140-6736(17)32336-X 28916366 PMC5603800

[4] Chao F You D Pedersen J Hug L Alkema L National and regional under-5 mortality rate by economic status for low-income and middle-income countries: a systematic assessment. Lancet Glob Health. 2018;6:e535-47. 10.1016/S2214-109X(18)30059-7 29653627 PMC5905403

[5] UNICEF Regional Office for South Asia. Reducing newborn mortality in South Asia: A results-based management approach to improving knowledge and accelerating results. UNICEF Regional Office for South Asia. 2016. Available: https://www.unicef.org/ rosa/reports/reducing-newborn-mortality-south-asia.

[6] Sharrow D Hug L You D Alkema L Black R Cousens S Global, regional, and national trends in under-5 mortality between 1990 and 2019 with scenario-based projections until 2030: a systematic analysis by the UN Inter-agency Group for Child Mortality Estimation. Lancet Glob Health. 2022;10:e195-206. 10.1016/S2214-109X(21)00515-5 35063111 PMC8789561

[7] Liu L Oza S Hogan D Chu Y Perin J Zhu J Global, regional, and national causes of under-5 mortality in 2000–15: an updated systematic analysis with implications for the Sustainable Development Goals. Lancet. 2016;388:3027-35. 10.1016/S0140-6736(16)31593-8 27839855 PMC5161777

[8] United Nations. World Population Prospects Department of Economic and Social Affairs, United Nations. 2019. Available: https://www.un.org/development/ desa/publications/world-population-prospects-2019-highlights.html.

[9] Billah SM Raihana S Ali NB Iqbal A Rahman MM Khan ANS Bangladesh: A success case in combating childhood diarrhoea. J Glob Health. 2019;9:020803. 10.7189/jogh.09.020803 31673347 PMC6816141

[10] Islam MM Islam MK Hasan MS Hossain MB Adolescent motherhood in Bangladesh: Trends and determinants. PLoS One. 2017;12:e0188294. 10.1371/journal.pone.0188294 29176807 PMC5703513

[11] Khan MN. Effects of Unintended Pregnancy on Maternal Healthcare Services Use in Bangladesh. Faculty of Health and Medicine, School of Medicine and Public Health, The University of Newcastle, Newcastle, Australia. 2020; 1.

[12] Tikmani SS Ali SA Saleem S Bann CM Mwenechanya M Carlo WAeditors. Trends of antenatal care during pregnancy in low-and middle-income countries: findings from the global network maternal and newborn health registry. Semin Perinatol. 2019;43:297-307. 10.1053/j.semperi.2019.03.020 31005357 PMC7027164

[13] Clyde P, Haig A, Jhaveri E, Karazja M, Leroueil P, Ranganathan K, et al. 25 Years of Health Care Delivery in Low-and Middle-Income Countries. William Davidson Institute at the University of Michigan, Article Series. 2019. Available: https://wdi.umich.edu/wp-content/uploads/WDI-25-Years-of-Health-Care-Delivery-in-Low-and-Middle-Income-Countries-WEB.pdf

[14] Decker MR Kalamar A Tunçalp Ö Hindin MJ Early adolescent childbearing in low-and middle-income countries: associations with income inequity, human development and gender equality. Health Policy Plan. 2017;32:277-82. 28207067 10.1093/heapol/czw121

[15] Glassman AL, Silverman R, McQueston K. Adolescent fertility in low-and middle-income countries: effects and solutions. Center for Global Development Working Paper. 2012. Available: https://www.cgdev.org/publication/adolescent-fertility-low-and-middle-income-countries-effects-and-solutions-working-paper

[16] Li Z Karlsson O Kim R Subramanian S Distribution of under-5 deaths in the neonatal, postneonatal, and childhood periods: a multicountry analysis in 64 low-and middle-income countries. Int J Equity Health. 2021;20:109-11. 10.1186/s12939-021-01449-8 33902593 PMC8077916

[17] Houweling TA Socio-economic inequalities in childhood mortality in low and middle income countries. Br Med Bull. 2010;93:7-26. 10.1093/bmb/ldp048 20007188

[18] Joarder T Chaudhury TZ Mannan I Universal health coverage in Bangladesh: activities, challenges, and suggestions. Psyche (Camb Mass). 2019;2019:4954095. 10.1155/2019/4954095 33281233 PMC7691757

[19] Khan JR Awan N A comprehensive analysis on child mortality and its determinants in Bangladesh using frailty models. Arch Public Health. 2017;75:58. 10.1186/s13690-017-0224-6 28912949 PMC5588744

[20] Mani K Dwivedi SN Pandey RM Determinants of under-five mortality in rural empowered action group states in India: an application of cox frailty model. Int J MCH AIDS. 2012;1:60. 10.21106/ijma.9 27621959 PMC4948162

[21] Yaya S Ahinkorah BO Ameyaw EK Seidu A-A Darteh EKM Adjei NK Proximate and socio-economic determinants of under-five mortality in Benin, 2017/2018. BMJ Glob Health. 2020;5:e002761. 10.1136/bmjgh-2020-002761 32843572 PMC7449341

[22] Mugo NS Agho KE Zwi AB Damundu EY Dibley MJ Determinants of neonatal, infant and under-five mortality in a war-affected country: analysis of the 2010 Household Health Survey in South Sudan. BMJ Glob Health. 2018;3:e000510. 10.1136/bmjgh-2017-000510 29527340 PMC5841513

[23] Rachmawati PD Kurnia ID Asih MN Kurniawati TW Krisnana I Arief YS Determinants of under-five mortality in Indonesia: A nationwide study. J Pediatr Nurs. 2022;65:e43-8. 10.1016/j.pedn.2022.02.005 35216837

[24] National Institute of Population Research and Training (NIPORT) and ICF. Bangladesh Health Facility Survey 2017. NIPORT,and ICF, Dhaka, Bangladesh.

[25] National Institute of Population Research and Training (NIPORT) and ICF. Bangladesh Demography and Health Survey, 2017/18. NIPORT,and ICF, Dhaka, Bangladesh.

[26] Burgert CR, Prosnitz D. 2014. Linking DHS Household and SPA Facility Surveys: Data Considerations and Geospatial Methods. DHS Spatial Analysis Reports No. 10. Rockville, Maryland, USA: ICF International. Available: https:// dhsprogram. com /pubs /pdf/SAR10/SAR10.pdf.

[27] World Health Organization. Neonatal mortality, infant mortality and under-five mortality. World Health Organization, Geneva, Switzarland. 2020. Available: https://www.who.int/ news-room/fact-sheets/detail/newborns-reducing-mortality.

[28] Tegegne TK Chojenta C Forder PM Getachew T Smith R Loxton D Spatial variations and associated factors of modern contraceptive use in Ethiopia: a spatial and multilevel analysis. BMJ Open. 2020;10:e037532. 10.1136/bmjopen-2020-037532 33046466 PMC7552846

[29] Chowdhury AH Hanifi SMA Bhuiya A Social determinants of under-five mortality in urban Bangladesh. J Popul Res. 2020;37:161-79. 10.1007/s12546-019-09240-x

[30] Majumder AK May M Pant PD Infant and child mortality determinants in Bangladesh: Are they changing? J Biosoc Sci. 1997;29:385-99. 10.1017/S0021932097003854 9881143

[31] Khan MA Khan N Rahman O Mustagir G Hossain K Islam R Trends and projections of under-5 mortality in Bangladesh including the effects of maternal high-risk fertility behaviours and use of healthcare services. PLoS One. 2021;16:e0246210. 10.1371/journal.pone.0246210 33539476 PMC7861360

[32] Rahman MS Rahman MS Rahman MA Determinants of death among under-5 children in Bangladesh. Journal of Research and Opinion. 2019;6:2294-302.

[33] Diez-Roux AV Multilevel analysis in public health research. Annu Rev Public Health. 2000;21:171-92. 10.1146/annurev.publhealth.21.1.171 10884951

[34] Zhongming Z, Linong L, Xiaona Y, Wangqiang Z, Wei L. Low quality healthcare is increasing the burden of illness and health costs globally. World Health Organization, Geneva, Switzarland. http://www.who.int/servicedeliverysafety/quality-report/en/

[35] Lungu EA Biesma R Chirwa M Darker C Healthcare seeking practices and barriers to accessing under-five child health services in urban slums in Malawi: a qualitative study. BMC Health Serv Res. 2016;16:410. 10.1186/s12913-016-1678-x 27542836 PMC4992285

[36] Alemu AT Walle AA Atnafu DD Quality of pediatric healthcare services and associated factors in Felege-Hiwot comprehensive specialized hospital, North-West Ethiopia: parental perception. Patient Prefer Adherence. 2020;14:1649-58. 10.2147/PPA.S264106 32982189 PMC7509332

[37] Ferdous F Azam AZ Utilization of child health care services in Thana Health Complex of Bangladesh: a study of Keraniganj. Asian J Epidemiol. 2009;2:20-32. 10.3923/aje.2009.20.32

[38] Leslie HH Spiegelman D Zhou X Kruk ME Service readiness of health facilities in Bangladesh, Haiti, Kenya, Malawi, Namibia, Nepal, Rwanda, Senegal, Uganda and the United Republic of Tanzania. Bull World Health Organ. 2017;95:738. 10.2471/BLT.17.191916 29147054 PMC5677617

[39] Narayanan I Nsungwa-Sabiti J Lusyati S Rohsiswatmo R Thomas N Kamalarathnam CN Facility readiness in low and middle-income countries to address care of high risk/small and sick newborns. Matern Health Neonatol Perinatol. 2019;5:10-4. 10.1186/s40748-019-0105-9 31236281 PMC6580648

[40] Monteiro S Shannon M Sandora TJ Chung S Pediatric aspects of hospital preparedness. Clin Pediatr Emerg Med. 2009;10:216-28. 10.1016/j.cpem.2009.06.001

[41] Islam A Biswas T Health system in Bangladesh: challenges and opportunities. Am J Health Res. 2014;2:366-74. 10.11648/j.ajhr.20140206.18

[42] Naher N Balabanova D McKee M Khan MH Roy P Ahmed SM Absenteeism among doctors in the Bangladesh health system: What are the structural drivers? SSM-Qualitative Research in Health. 2022;2:100089. 10.1016/j.ssmqr.2022.100089

[43] Riaz BK Ali L Ahmad SA Islam MZ Ahmed KR Hossain S Community clinics in Bangladesh: a unique example of public-private partnership. Heliyon. 2020;6:e03950. 10.1016/j.heliyon.2020.e03950 32420500 PMC7218291

[44] Karra M Fink G Canning D Facility distance and child mortality: a multi-country study of health facility access, service utilization, and child health outcomes. Int J Epidemiol. 2017;46:817-26. 27185809 10.1093/ije/dyw062

[45] Kadobera D Sartorius B Masanja H Mathew A Waiswa P The effect of distance to formal health facility on childhood mortality in rural Tanzania, 2005–2007. Glob Health Action. 2012;5:1. 10.3402/gha.v5i0.19099 23151364 PMC3495250

[46] Sarrassat S Meda N Badolo H Ouedraogo M Somé H Cousens S Distance to care, care seeking and child mortality in rural Burkina Faso: findings from a population-based cross-sectional survey. Trop Med Int Health. 2019;24:31-42. 10.1111/tmi.13170 30347129 PMC6378618

[47] Zaman SM Cox J Enwere G Bottomley C Greenwood B Cutts F The effect of distance on observed mortality, childhood pneumonia and vaccine efficacy in rural Gambia. Epidemiol Infect. 2014;142:2491-500. 10.1017/S0950268814000314 24565180 PMC9151291

[48] Yu JC Khodadadi H Malik A Davidson B Salles ÉdSL Bhatia J Innate immunity of neonates and infants. Front Immunol. 2018;9:1759. 10.3389/fimmu.2018.01759 30105028 PMC6077196

[49] Khan MN Islam MM Effect of exclusive breastfeeding on selected adverse health and nutritional outcomes: a nationally representative study. BMC Public Health. 2017;17:889. 10.1186/s12889-017-4913-4 29162064 PMC5697409

[50] World Bank. Current Health Expenditure. World Bank, New York, USA. 2017. Available: https://data.worldbank.org/indicator/SH.XPD.CHEX.GD.ZS?locations=BD (% of GDP).

[51] Sato RKM, Elewonibi B, Mhande M, Msuya S, Shah I, Canning D. Distance to Facility and Healthcare Utilization in Tanzania: Unbiased and Consistent Estimation using Perturbed Location Data. Population Association of America, New York, USA. 2019. Available: http://paa2019.populationassociation.org/uploads/193166

