# Supplementary material for: Availability and readiness of health care facilities and their effects on under-five mortality in Bangladesh: Analysis of linked data
Source: J Glob Health. 2022 Sep 17;12:04081. doi: 10.7189/jogh.12.04081 (PMC9480612; doi:10.7189/jogh.12.04081)
Supplement: Online Supplementary Document [file jogh-12-04081-s001.pdf]

ONLINE SUPPLEMENTARY DOCUMENT

Title: Availability and readiness of healthcare facilities and their effects on under-five mortality in Bangladesh: Analysis of linked data

Authors: Md Nuruzzaman Khan, Nahida Islam Trisha, Md. Mamunur Rashid

Supplementary Table S1: Variables included in creating the health facility level factors

Name of health facility level factors	Number of items	Variables considered in generating health facility level factors
Basic management and administrative system of the nearest healthcare facility where child healthcare services are available	7	**Supportive management for providers for child health services:** availability of healthcare personnel who received training related to child health during the 24 months preceding the survey, availability of healthcare personnel who received training related to personal supervision during the 6 months preceding the survey, and availability of healthcare personnel who received training related to child health during the 24 months and personal supervision during the 6 months preceding the survey.<br><br>**Training for child health service providers in the past 24 months:** EPI/cold chain, IMCI, ARI, and diarrhoea diagnosis
Degree of availability of child healthcare services at the nearest healthcare facility	35	**Child health services:** outpatient curative care for sick children, growth monitoring, child vaccination services, and diagnosis of and/or treatment for child nutrition.<br><br>**Equipment for child curative care services:** child scale, length or height broad, thermometer, stethoscope, infant scale, growth chart, MUAC tape, timer.<br><br>**Trained staff for child curative care services:** IMCI (during the past 24 months), growth monitoring (during the past 24 months)<br><br>**Essential and priority medicine:** ORS, amoxicillin syrup, suspension, or dispersible, cotrimoxazole syrup, suspension, or dispersible, paracetamol syrup or suspension, vitamin A capsules, mebendazole/albendazole, zinc tablets or syrup, ampicillin powder for injection, ceftriaxone powder for injection, gentamycin for injection, and benzathine benzylpenicillin for injection<br><br>**Infection control and laboratory diagnostic capacity:** soap, running water, alcohol-based hand disinfectant, latex gloves,

		sharps container, waste receptacle, haemoglobin test, stool microscopy.
Readiness of the mothers' homes nearest healthcare facility (where child healthcare services are available) to provide child healthcare services	10	IMIC guidelines, staff trained in IMIC, child scale, thermos-meter, growth chart, zinc tablets or syrup, ORS, amoxicillin syrup, suspension, or dispersible, paracetamol syrup or suspension, meben-dazole/alben-dazole.
Average distance on road communication from mothers' resided cluster to the nearest healthcare facility where child healthcare services are available		Average distance on road communication from women's resided cluster to the nearest health facility.
